# Erosion and Sediment Transport Modelling in Shallow Waters: A Review on Approaches, Models and Applications

**DOI:** 10.3390/ijerph15030518

**Published:** 2018-03-14

**Authors:** Mohammad Hajigholizadeh, Assefa M. Melesse, Hector R. Fuentes

**Affiliations:** 1 Department of Civil and Environmental Engineering, Florida International University, 10555 W Flagler Street, EC3781, Miami, FL 33174, USA; 2 Department of Earth and Environment, Florida International University, AHC-5-390, 11200 SW 8th Street Miami, FL 33199, USA; melessea@fiu.edu; 3 Department of Civil Engineering and Environmental Engineering, Florida International University, 10555 W Flagler Street, Miami, FL 33174, USA; fuentes@fiu.edu

**Keywords:** erosion and sediment transport, mathematical and computer models, types of models, classification, applications, shallow waters

## Abstract

The erosion and sediment transport processes in shallow waters, which are discussed in this paper, begin when water droplets hit the soil surface. The transport mechanism caused by the consequent rainfall-runoff process determines the amount of generated sediment that can be transferred downslope. Many significant studies and models are performed to investigate these processes, which differ in terms of their effecting factors, approaches, inputs and outputs, model structure and the manner that these processes represent. This paper attempts to review the related literature concerning sediment transport modelling in shallow waters. A classification based on the representational processes of the soil erosion and sediment transport models (empirical, conceptual, physical and hybrid) is adopted, and the commonly-used models and their characteristics are listed. This review is expected to be of interest to researchers and soil and water conservation managers who are working on erosion and sediment transport phenomena in shallow waters. The paper format should be helpful for practitioners to identify and generally characterize the types of available models, their strengths and their basic scope of applicability.

## 1. Introduction

Soil erosion and its degradation of soil productivity and environment effects on the productivity of land and water quality (of rivers, estuaries and lakes) comprise one of the major concerns of watershed managers and decision makers. Temporal and spatial information of soil erosion processes is required to reflect the pattern of sediment transport during storm events. Erosion is a process of detachment and transport of soil materials by erosive agents from any part of the Earth’s surface [1]. Generally, natural erosion is divided into two main categories: water erosion and wind erosion. Water erosion occurs as different forms of splash, sheet and interrill erosion, rill erosion, gully erosion, river banks or channel erosion, tillage erosion and glacial erosion. Factors affecting water erosion are climate, topography, soil structure, vegetation and anthropogenic activities such as tillage systems and soil conservation measures [2]. Sheet and interrill erosion is considered one of the first steps of erosion in catchments, which is widely observed on bare or almost bare soils in agricultural lands, pasturage and open areas. In this type of erosion, the process begins by rain drops hitting the soil surface, and their effect of detaching the soil structure is an important factor in particulate matter transport. Generally, rainfall intensity and runoff rate are the major determinants of splash and sheet erosion [3]. Particularly in very shallow water, raindrops can provide temporary disturbances that cause static particles to move. Subsequently, the overland flow transports the sediment in a downslope direction. Three types of transportation are taking place in sheet erosion: raindrop splash, overland flow action and the combination of overland flow and rainfall impact [4]. However, the main cause of erosion on steep slopes or any area with sparse land cover is concentrated flow [5], and flow hydraulic parameters and transport capacities determine the concentrated flow erosion rates [6].

Due to the increasing use of computer applications and computing power in recent decades, the investigations of soil erosion and sediment transport through the development of computer models have been rapidly increased, developed and provided new possibilities. However, there are still many models that suffer from a range of problems, such as over-estimation due to the uncertainty of models and the unsuitability of assumptions and parameters in compliance with local conditions. Over-parameterization due to the deficiency of testing the model is also an issue. The key objective of this paper is to provide a source that addresses these processes with details for the researchers who are involved in studying the transport process of sediment by overland flow and shallow waters. This scope could be achieved by reviewing a number of existing models and studies in the mentioned field, the knowledge and concepts behind these models and the characteristics of the models including their inputs-outputs.

Many physics-based algorithms have been developed recently to describe the processes of detachment and sediment transport by shallow overland flow. These algorithms commonly have been inspired by the state sediment flux equation [7], the fundamental energy transport equation [8] and the steady state continuity equation for rill and interrill detachment and/or deposition [9]. Sediment transport capacity concepts and relationships, which initially were developed for channels and alluvial rivers, are adopted for use in shallow water flows, and different complexities are widely used in these algorithms. Indeed, most of the mathematical models of soil erosion in shallow waters are borrowed from the field of fluvial sediment transport [10]. There are significant differences between shallow overland flow and deeper channel flow [11]. However, knowledge of the shallow overland flow hydraulics and soil erosion mechanics have been increasing recently, but little research has been published explaining the physical mechanisms of particulate matter wash-off in shallow flow. 

**Figure 1:**
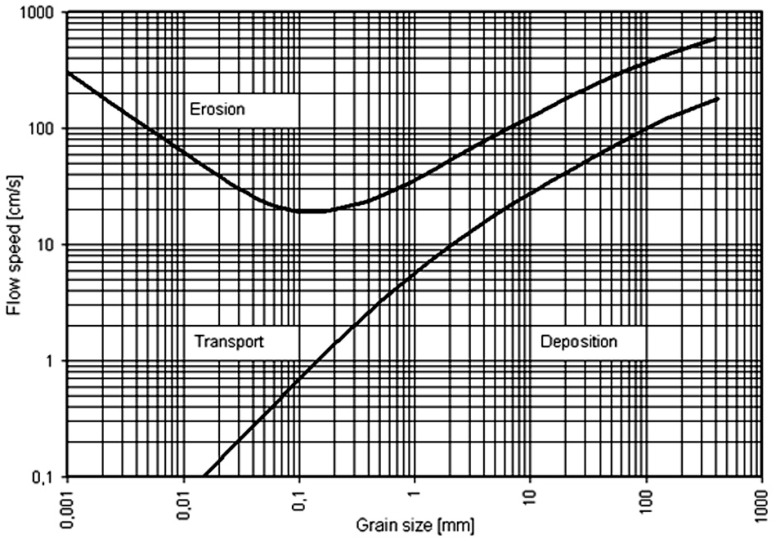
The Hjulstrom diagram. Source: Wikipedia.

As mentioned, sediment transport capacity is a major concept to determine the rates of detachment and deposition in physically-based erosion and sediment transport models. In most of the physically-based models, the erosion process is divided into concentrated flow (rill) erosion and splash and sheet (inter-rill) erosion [7,12,13,14]. The transportability of sediment by overland flow depends on the sediment concentration. During severe rainfall events or high intensity rainfalls, sediment concentration is higher compared to lower rainfall intensity. This is due to the greater power of rainfall in triggering the detachment of soil particles. On the other hand, by increasing the flow depth, sediment concentration decreases and causes the transport capacity to be increased again. Hjulstrom [15] developed a graph to show the relationship between the size of sediments and the velocity required to erode (lift it), transport and deposit the soil particles (Figure 1).

Proffitt [16] expressed that the detachability or re-detachability, and thereby, the amounts of soil loss, is expected to decrease when the overland flow depth is increased. Many laboratory experiments have provided the necessary knowledge to establish better relationships between different hydraulic parameters and sediment transport capacity in shallow waters. This information is the initial component for any physically-based erosion and sediment transport models. For more complex problems involved in the concurrent processes of erosion and sediment transport in non-uniform flows on varying topography or other situations that provide unsteady flows, numerical solutions are required in these models. In situations with simpler scenarios and when assumptions are made, the model can be analytically solved.

Over the past few decades, remote sensing and geographic information systems (GIS) have been widely used to develop spatially-distributed models of watershed hydrological processes for automated extraction of watershed structure from digital elevation model (DEM), land use/land cover, soils, vegetation maps, etc. [17,18,19,20,21]. Much information such as canopy leaf area index, slope, aspect, contributing drainage area, soil texture or hydraulic conductivity assigned by soil series, and so on, can be automatically imported into the models using remote sensing and GIS techniques. 

The structure of this paper is to review most of the available literature concerning sediment transport modelling in shallow waters and to make a classification based on the representational process of the model adopted, and the commonly-used models and their characteristics are listed. Model types are categorized in terms of how the processes of soil detachment, transport and deposition are represented by the model. It provides descriptions of a number of available models that are widely used in the market. The review is expected to be of interest to researchers, decision makers and water quality managers who are concerned with erosion and sediment transport phenomena in shallow waters. This paper will conclude with the major issues of the introduced erosion and sediment transport models, including discussions about the models’ complexity and accuracy, data availability and models’ uncertainties. This review is prepared to provide an overview of the wide range of issues related to the erosion and sediment transport processes in shallow waters. For a detailed analysis of these components, the reader is required to refer to the appropriate references throughout this text prior to modelling.

## 2. Soil Erosion and Sediment Transport Models

A wide range of soil erosion models has been developed in the past few decades, each differing in terms of complexity, accuracy, inputs and outputs, approaches and their spatial and temporal scales. Generally, based on the physical processes simulated by the model, approaches to generate the data and data dependence, different kinds of models can be categorized into four widely-used models including:

(1) Empirical models,
(2) Conceptual models,
(3) Physically-based models,
(4) Hybrid models.

The accuracy of soil erosion measurement depends on model type and the considered parameters. For example, Kinnell [22,23] pointed out that both conceptual and empirical models have some inadequacy in characterizing the soil loss in comparison to observed erosion values in bare soils. Models may also be described as hybrids between two or more of these classes. For example, the Identification of unit Hydrographs And Component flows from Rainfall, Evaporation and Streamflow-Water Quality (IHACRES-WQ) model [24] and European SEDiment NETwork (SEDNET) model [25] are hybrid metric-conceptual models. The structure of the models consists of a number of storages and is basically conceptual, while statistical identification procedures are used to determine the number and configuration of storages in each catchment [9]. 

Most of the studies are performed on bare soils and in some cases on tilled soils covered by grass or mulch. However, natural systems are more complex and represent many variations in terms of spatial and temporal scales, transport media and dimensions and the interactions between detached sediment and attached chemicals. Jakeman et al. [26] stated that environmental modeling is limited by natural complexity, spatial heterogeneity and the lack of available data. As an example, in forested areas, high variability in the spatial and temporal distribution of vegetation and soil properties may be seen. In such areas, different types of surface cover, runoff-generating mechanisms and various spatial and temporal patterns of hydraulic conductivity, infiltration capacity and surface erodibility are experienced [27,28,29,30,31,32,33,34,35,36,37,38,39,40]. These factors can cause different values of sediment generation and deposition.

It should be noted that it is impossible to choose the ‘best’ model among those available, because each model has been developed for a particular purpose and is unable to solve the problem in every situation. A number of factors that should be considered in order to choose an appropriate model for a particular purpose include:

- Dataset requirements of the model,
- Fundamental assumptions of the model,
- The accuracy and validity of the model,
- Model capabilities and susceptibilities,
- The components of the model,
- User-friendliness of the model,
- The objectives of the model,
- The scales of the model outputs,
- Hardware requirements of the model.

### 2.1. Empirical Models 

Empirical models are a simulation of natural processes, mostly based on statistical observations, and rely on developed regression relationships. The computational processes of empirical models are simple, and their data requirements are less than those that are required for conceptual and physically-based models. In this way, it can be said that empirical models are the simplest approach to measure soil erosion and sediment transport when compared to the other three types of models. The difficulty with using empirical models is the inability to be accurately used outside of the geographical area where their relationships were derived. Empirical models also may utilize unrealistic assumptions about the physics of the catchment system and, therefore, ignore the heterogeneity of some catchment inputs such as rainfall and soil types. In addition, it should be noted that the inherent non-linear relations in the catchment system are ignored in empirical models [41]. 

Morgan [42] introduced three types of analyses for empirical models: (1) black-box analysis, where only the main inputs and outputs are studied; (2) grey-box analysis, where the system’s procedure is explained in more detail; and (3) white-box analysis, where the elements of the system are represented in detail. An advantage of empirical models is their ability to be employed in catchments with limited data, and also the lack of a requirement for complex inputs; thus, they can be considered preferable to more complex conceptual and physically-based models. Empirical models are valuable as a first step in identifying sources of sediment and nutrient generation. At regional scales, with the recognition of sediment residence time and delivery patterns, empirical methods can be applied uniformly to predict the sediment delivery [25]. In the empirical models, the parameter values can be obtained from local calibrations, although sometimes transferred from calibrations at experimental sites [9]. 

The first investigations resulted in the development of the following empirical models: Sediment Rating Curve [43], Musgrave Equation [44], Dendy–Bolton Method Flaxman Method [45,46], Sediment Delivery Ratio Method [47], Runoff-Sediment Yield Relation [48,49]. Table 1 represents the list of commonly-used empirical models with their characteristics and sources.

### 2.2. Conceptual Models

Conceptual models are basically a combination of empirical and physically-based models and are more applicable to answering general questions [63]. These models were developed on the basis of spatially-lumped forms of water and the sediment continuity equation [64]. The main focus of a conceptual model is to predict sediment yield, basically using the concept of the unit hydrograph. Conceptual models represent a catchment by its internal storage systems, which typically incorporate the inherent physical processes of runoff generation and sediment transport in their conceptual structure. These models usually unify general descriptions of catchment processes without specifying the process of interactions that would require very detailed catchment information [65]. These models therefore provide an indication of the quantitative and qualitative effects of land use changes within a watershed, without taking into consideration the data that are obtained from spatial and temporal input. The value of each parameter in conceptual models is obtained through calibration against observed data, such as stream discharge and sediment concentration measurements [66]. Therefore, due to this requirement, conceptual models tend to suffer from the identifiability problems of their parameter values [67]. Generally, simple conceptual models have fewer problems with model identification than more complex models. Thus, to minimize the problems with model identification, the number of parameters to be estimated through calibration can be reduced where applicable [41,68]. However, this simplification of models may affect the goodness of fit to calibration data. 

The first investigations to derive conceptual models led to the development of the following models: Sediment Concentration Graph [69], Renard–Laursen Model [70], Unit Sediment Graph [71], Instantaneous Unit Sediment Graph [72], Sediment Routing Model [73], Discrete Dynamic Models [74], Muskingum Sediment Routing Model [75]. The list of commonly-used conceptual models and their characteristics and sources are summarized in Table 2.

## 3. Physically-Based Models 

Physically-based models are generally based on the concept of the conservation of mass, momentum equations and energy as governing equations describing streamflow or overland flow, and conservation of mass equation for sediment [98,99]. Most of the developed physically-based soil erosion models that are being used worldwide to predict erosion and sediment yield are not 100% physically-based because mathematical expressions describing each individual process are developed based on the empirical/conceptual approaches and their assumptions and consideration [100]. Physically-based models, in particular, are often over-parametrized [41,101]. Basically, the parameters of physically-based models are independently measurable. However, due to the existence of a large number of complex parameters and the heterogeneity of critical characteristics, especially in catchments, calibration of these parameters with observed data is inevitable [41]. This procedure creates extra uncertainties in parameter values. In this situation, with the large number of parameter values (in some cases, hundreds) that are required to be measured through the mentioned process, the ability to identify the model parameters will become very difficult, and the non-uniqueness of ‘best fit’ solutions can be expected [63]. 

Generally, the governing equations in physically-based models are derived at a small scale and under very specific physical conditions. However, in many cases, these equations are regularly applied to a greater scale with different physical conditions. Continuous spatial and temporal data have been considered for use in these equations, although it is used most often in practice point source data taken to represent an entire grid cell in the catchment. This manner of scaling up is questionable [102], as these small-scale parameters that are assumed for application in small-scale models have the potential to lose their physical significance when they are applied to larger scales [103]. There is not enough theoretical justification to assume that equations can be used identically at the grid scales that represent the lumped aggregate of heterogeneous sub-grid processes [9]. The mathematical expressions in physically-based models, which are derived to describe individual processes, have many assumptions that may not be relevant in most of the natural conditions [104]. Beven [105] notes that by calibration-based model parameterization, physical distributed models are equal to any conceptual model.

The Erosion Kinematic Wave Models [106,107,108], Quasi-Steady State Erosion [109], ANSWERS (Areal Non-point Source Watershed Environment Response Simulation) [110], CREAMS (Chemical Runoff and Erosion from Agricultural Management Systems) [111] and Continuum Mechanics Model [112] are among the first examples of researchers’ efforts to develop physically-based models. Table 3 represents the list of commonly-used physically-based models with their characteristics and sources.

### Hybrid Models

Hybrid models are a mixture of dynamic and empirical soil erosion evaluation techniques. The structure of hybrid models is usually physical or conceptual at the core, while the configuration of the model in the spatial and temporal scales is based on statistical observations and relies on developed regression relationships. For example, in an empirical-conceptual hybrid model, the structure is conceptualized as a set of storages, and effective rainfalls are modelled at these scales to generate runoff values. In the empirical phase, the statistical identification procedure is applied to determine the metric component of the model, the storage number and configurations per catchment.

Hybrid models developed as soil erosion and sedimentation modelling systems can be used to predict the water erosion vulnerability, soil productivity reduction at hillslopes, catchments and farms and can also assess the optimal management strategies for agricultural or soil and water conservation practices. The list of commonly-used hybrid models and their characteristics and sources are summarized in Table 4.

## 4. Criteria for the Selection of a Proper Model for Study

Most of the soil erosion and sediment yield and transport models have their own capabilities and limitations based on their complexities, uncertainties, data availability and accuracy, objectives and spatial and temporal scales of study. There are still many difficulties in the understanding and description of event-based procedures that cause erosion, and this may lead to exaggeration of the erosion and sediment yield when combined with the insufficiency and inaccuracy in the interpretation of data. Due to the limitations and difficulties in fully understanding the complexity of natural systems, especially over large watersheds, empirical models are more widely used than physically-based models to solve specific problems in large-scale ecosystems. Furthermore, the large database required by physically-based models is not always easily accessible and available for all watersheds, especially in developing countries.

Many successes and failures derived from different widely-used models that are reported in the literature should be considered precisely by modelers and the agencies that support the models’ development to promote and enhance the usefulness of existing models. Simulated results should be validated and calibrated by comparing with the field-measured data. However, the limitations, capacities and capabilities of the model, sensitivities, uncertainties, assumptions, required inputs and expected results, license costs and other determined physiographic and climatic conditions of models must be studied and considered by users. Based on these criteria, to select a proper model to obtain the desired results, a user should first know what the simulation outputs of the model are. Furthermore, special attention should be given to the spatial (field, catchment, hillslope and regional) and temporal (continuous, event, daily, monthly, seasonal and annual) scales of the model, which are important factors in the selection of a proper tool for a specific study. It is highly recommended to use accredited and validated models that have been previously used to simulate erosion and sediment processes in similar physiographic and climatic conditions. In addition, accurate data are required to obtain reliable results from erosion and sediment prediction models. 

## 5. Conclusions 

Soil erosion caused by water as a natural phenomenon appears in different types and has direct and indirect effects on the environment and human life. It reduces the productivity of lands and decreases the useful storage volume of rivers and reservoirs and the service life of many hydraulic structures, like dams, by deposition of sediments. During the past few decades, a large number of soil erosion and sediment transport models has been developed, focusing on various characteristics and capacities. Based on their underlying concept, these models are categorized into four groups: (I) empirical models, (II) conceptual models, (III) physically-based models and (IV) hybrid models. 

Among the empirical models, the Universal Soil Loss Equation (USLE) model is widely described in the literature. Although it was mainly developed based on data from the United States, this model and its latter Revised (RUSLE) and Modified (MUSLE) versions are widely applied all around the world with a large number of subsequently developed models based on this model. 

Hydrologic simulation Program, Fortran (HSPF), is considered as a conceptual model that is freely available. It is suitable for large watersheds comprised of both urban and rural areas. HSPF can address the sediment and nutrient Total Maximum Daily Load (TMDL) problems, nutrient and pesticide management, urbanization and ponds. It calculates the amounts of deposition or scour of cohesive sediment based on the bed shear stress. The critical shear stress required for the calculation of deposition and scouring is determined by the user, and deposition or scouring of cohesive bed sediments occurs whenever shear stress is less than or greater than the specified critical shear stress, respectively. The simplified Krone’s equation [156] is used in this model to measure the rate of deposition based on settling velocity, sediment concentration, shear stress and critical shear stress [157]. The Soil and Water Assessment Tool (SWAT) model is another conceptual model that is continuously under development and largely used worldwide. SWAT is a regional-scale and continuous-time model, which operates on a daily time step at the basin scale. It can be used to predict both overland and in-stream sediment generation, transportation and deposition, as well as rainfall-runoff and sediment-associated transport of chemicals. Prediction of the long-term impacts of erosion in large basins, as well as the timing of agricultural practices within a year are other applications of this model that cause it to be used frequently. Physically-based models are developed on the basis of the physical description of the soil erosion and sediment transport processes. Working with these models is more complex than other models due to their highly detailed representations of the processes and the requirement of the preparation of extensive data. These issues have caused models such as Limburg Soil Erosion Model (LISEM) and Watershed Erosion Prediction Project (WEPP) to be less frequently adopted [9] and has led to efforts to develop physically-based erosion and sediment transport models that are simplified and require less data.

However, physically-based models are more capable of operating either on a continuous basis or in an event-based mode, like MIKE-Systeme Hydrologique Europeen (SHE), CASCade 2-Dimentional SEDimentation (CASC2D-SED), Watershed Erosion Prediction Project (WESP), SEM, SHE-SEDimentation (SHESED) and EUROpe WIthin Storm Erosion (EUROWISE). The Danish Hydraulic Institute (DHI)’s MIKE-SHE is a physically-based watershed model, which also contains several Best Management Practices (BMPs) options, such as wetlands, nutrient and pesticide management. For river hydraulics purposes, MIKE-SHE can be used with MIKE-11. Groundwater Loading Effects of Agricultural Management Systems (GLEAMS) is another model that is used for hydrology, water quality and nutrient analysis, pesticide transport and erosion and sediment yield for field-scale agricultural areas. In this model, the USLE computation methods are used and implemented to measure the rates of erosion and sediment yield. The Kinematic Runoff and Erosion Model-2 (KINEROS-2) is the improved version of KINEROS, which is an event-based model suitable to analyze surface runoff and erosion rates over small natural and urban watersheds. KINEROS-2 can consider both concentrated flow (rill) erosion due to flowing water and splash and sheet (inter-rill) erosion resulting from raindrop energy, separately. This model also can prepare the input data and visualize the results is a GIS format. An important disadvantage of this model is its lack of considering the Evapotranspiration (ET) losses. Furthermore, true soil moisture redistribution for long rainfall intervals cannot be formulated in KINEROS-2. 

GSSHA (Gridded Surface/Subsurface Hydrologic Analysis) is a 2D physically-based model, which simulates surface and groundwater hydrology. In previous versions of the model, the erosion and sediment component were semi-empirical; however, in recent versions, the sediment transport formulation is based on the USLE soil parameters. There are also different optional methods to simulate erosion and sediment transport, especially using a specific gravity different from sand. Other optional equations to calculate sediment transport include: Kilinc and Richardson [158], Englund Hanson [8] and Stream power [159]. GSSHA inputs can be driven by land use, soil, vegetation and other physiographic maps in GIS format, and it also links the model results with GIS.

DWSM, the Dynamic Watershed Simulation Model, is a storm event, distributed and physically-based model for simulations of surface and ground water flow, soil erosion and transport of sediment and chemicals (nonpoint-source pollutants) in a watershed during a single or a series of rainfall events. DWSM computes the rates of erosion based on the detachability of user-defined soil particles by raindrop impact and also erodibility of soil by flow characteristics. The sediment transport component and the process of scouring and deposition are computed based on the sediment transport capacity of flow using the approximate analytic solution of the temporally- and spatially-varying continuity equation. Drainage patterns and topographic features are considered to delineate the sub-watersheds. 

Hybrid models, which apply both metric and physical processes of soil erosion and sedimentation modelling systems, can be used to predict the water erosion vulnerability and the soil productivity reduction at hillslopes, catchments and farms and are also used to assess the optimal management strategies for agricultural or soil and water conservation practices. IHACRES-WQ, SEDNET, THORNES and Automated Geospatial Watershed Assessment (AGWA), in terms of their characteristics and their outputs, can be mentioned as the strongest models among the hybrid models. The determination of an appropriate model depends on the questions and problems that need to be addressed. Furthermore, the spatial and temporal scales, suitability, accuracy and validity of a model in catchment conditions, model assumptions and data requirements should be considered by the user. 

## Figures and Tables

**Table 1:** Empirical soil erosion models.

Model	Name	Spatial Scale	Temporal Scale	Data Demand	Output	Overland Sediment			In-Stream Sediment			Gully Erosion	Rainfall-Runoff	Sediment-Associated Chemicals	Source
						Gen. *	Trans. *	Dep. *	Gen. *	Trans. *	Dep. *				
PSIAC	Pacific Southwest Interagency Committee Method	Catchment, Field	Annual	High	sediment yield	No	No	No	No	No	No	Yes	Yes	No	[50]
MUSLE	Modified Universal Soil Loss Equation	Hillslope	Annual	High	Erosion	Yes	No	No	No	No	No	No	No	No	[51]
USLE	Universal Soil Loss Equation	Hillslope	Annual	High	Erosion	Yes	No	No	No	No	No	No	No	No	[52]
SLEMSA	Soil Loss Estimation Model for Southern Africa	Catchment	Annual	High	Soil loss, sheet erosion	Yes	No	No	No	No	No	No	No	No	[53]
RUSLE	Revised Universal Soil Loss Equation	Hillslope	Annual	High	Erosion	Yes	No	No	No	No	No	No	No	No	[54,55]
EPM	Erosion Potential Method	Catchment, Field	Annual	High	Erosion, sediment yield	Yes	No	No	No	No	No	Yes	Yes	No	[56]
SEDD	Sediment Delivery Distributed	Hillslope, Catchment	Annual, Event	High	Erosion, sediment yield	Yes	No	No	No	No	No	No	Yes	No	[57]
TCRP	Tillage-Controlled Runoff Pattern model	Field	Abstract	Low	Gully formation, runoff	No	No	No	No	No	No	Yes	Yes	No	[58]
MOSES	Modular Soil Erosion System project	Hillslope	Annual	High	Erosion, sediment yield, runoff	Yes	No	No	No	No	No	No	Yes	No	[59,60]
TMDL	Total Maximum Daily Load	Catchment	Annual	Low	Sediment	No	No	No	No	No	No	No	No	Yes	[61]
BQART	Not Found	Global/Regional	Annual	High	Erosion, sediment yield, runoff	Yes	Yes	Yes	Yes	Yes	Yes	No	No	No	[62]

Note: Gen. * = Generation; Trans. * = Transportation; Dep. * = Deposition.

**Table 2:** Conceptual soil erosion models.

Model	Name	Spatial Scale	Temporal Scale	Data Demand	Output	Overland Sediment			In-Stream Sediment			Gully Erosion	Rainfall-Runoff	Sediment-Associated Chemicals	Source
						Gen. *	Trans. *	Dep. *	Gen. *	Trans. *	Dep. *				
TOPMODEL	TOPMODEL	Hillslope	Daily	Medium	Sediment yield, runoff	No	No	No	No	No	No	No	Yes	Yes	[76]
HSPF	Hydrologic simulation Program, Fortran	Catchment	Daily	High	Sediment load, runoff, flow rate, nutrient	Yes	Yes	Yes	Yes	Yes	Yes	Yes	Yes	Yes	[77]
EPIC	Erosion-Productivity Impact Calculator	Field	Daily	High	Sediment load, nutrient	Yes	Yes	Yes	Yes	No	No	No	No	Yes	[78]
AGNPS	Agricultural Non-Point Source pollution model	Catchment	Daily	High	Erosion, sediment yield, runoff, peak rate, pollutants	Yes	No	No	Yes	Yes	Yes	Yes	Yes	Yes	[79]
SWAT	Soil and Water Assessment Tool	Regional	Daily	Medium	Erosion, sediment yield, runoff, peak rate, nutrient	Yes	Yes	Yes	Yes	Yes	Yes	No	Yes	Yes	[80,81]
SWRRB	Simulator for Water Resources in Rural Basins	Catchment	Daily	High	sediment, streamflow, nutrient and pesticide yields	Yes	No	No	Yes	Yes	Yes	No	Yes	Yes	[82]
ACRU	Agricultural Catchment Research Unit	Catchment	Daily	Low	Erosion, sediment yield, runoff, flow rate	Yes	No	No	Yes	No	No	Yes	Yes	Yes	[83]
APSIM	Agricultural Production Simulator	Field	Daily	High	Erosion, nutrient	Yes	Yes	Yes	No	No	No	No	Yes	Yes	[84]
SWIM	Soil and Water Integrated Model	Regional	Daily	Medium	Sediment load, runoff, nutrient	Yes	No	No	No	No	No	No	Yes	Yes	[85]
IQQM	Integrated Water Quality and Quantity Model	Regional	Daily	Medium	Sediment load, suspended sediments, pollutants transport, salt fluxes	Yes	No	No	Yes	No	No	No	Yes	Yes	[86]
RillGrow 1 and 2	RillGrow 1 and 2	Plot	Abstract	High	Rill formation	Yes	Yes	Yes	No	No	No	Yes	No	No	[87]
MEDRUSH	MEdalus Desertification Response Unit SHe	Catchment	Hourly	High	sediment transport, net erosion, runoff, soil moisture profiles	Yes	Yes	Yes	Yes	Yes	Yes	Yes	Yes	Yes	[88]
LASCAM	Large Scale Catchment Model	Catchment	Daily	High	Sediment load, runoff, salt fluxes	Yes	Yes	Yes	Yes	No	No	No	No	No	[89]
AGNPS-UM	Agricultural Non-Point Source pollution model, modified	Catchment	Daily	High	Suspended sediments, runoff, nutrient	Yes	No	No	Yes	Yes	Yes	Yes	Yes	Yes	[90]
EMSS	Environmental Monitoring Support System	Catchment	Daily	Low	Sediment load, runoff, nutrient	No	No	No	Yes	Yes	Yes	Yes	Yes	Yes	[91]
SEDNET	European SEDiment NETwork	Catchment	Steady-State	Moderate	Suspended sediment, sediment distribution, overland flow	Yes	No	No	No	No	No	No	Yes	Yes	[25]
STREAM	Sealing, Transfer, Runoff, Erosion, Agricultural Modification model	Catchment	Event	High	Erosion, sediment generation and transport	Yes	Yes	Yes	Yes	No	No	No	No	No	[92]
SERAE	Soil Erosion Risk Assessment in Europe model	Regional	Annual	Medium	Erosion	Yes	No	No	No	No	No	No	Yes	Yes	[93]
CAESAR	Cellular Automaton Evolutionary Slope and River model	Catchment-Regional	Annual	High	Erosion, sediment transport	Yes	Yes	Yes	Yes	Yes	Yes	Yes	Yes	No	[94]
WILSIM	Web-based Interactive Landform Simulation Model	Hillslope	Abstract	High	Erosion, sediment transport	Yes	No	No	Yes	No	No	No	No	No	[95]
INCA-C	Integrated Catchments Model for Carbon	Catchment	Daily	Daily	Suspended sediments, runoff, Dissolved Organic Carbon (DOC)	Yes	Yes	Yes	Yes	Yes	Yes	No	Yes	Yes	[96]
PSYCHIC	Phosphorus and Sediment Yield Characterization in Catchments	Catchment	Daily	High	Sediment load, nutrient	Yes	Yes	Yes	No	No	No	No	Yes	Yes	[97]

Note: Gen. * = Generation; Trans. * = Transportation; Dep. * = Deposition.

**Table 3:** Physically-based soil erosion models.

Model	Name	Spatial Scale	Temporal Scale	Data Demand	Output	Overland Sediment			In-Stream Sediment			Gully Erosion	Rainfall-Runoff	Sediment-Associated Chemicals	Source
						Gen. *	Trans. *	Dep. *	Gen. *	Trans. *	Dep. *				
ANSWERS	Areal Nonpoint Source Watershed Environment Response Simulation	Small Catchment	Event	High	Erosion, sediment yield, runoff, peak rate, nutrients	Yes	Yes	Yes	No	No	No	No	Yes	Yes	[110]
CREAMS	Chemicals, Runoff and Erosion from Agricultural Management Systems	Field/ Plot	Monthly	High	Erosion, deposition	Yes	Yes	Yes	No	No	No	Yes	Yes	Yes	[111]
SPNM	Sediment–Phosphorus–Nitrogen Model	Hillslope/ Catchment	Event	High	Erosion, sediment yield, runoff, nutrients	No	No	No	Yes	Yes	Yes	No	Yes	Yes	[113]
TOPOG	The Terrain Analysis Hydrologic Model	Hillslope/ Catchment	Daily	High	Erosion hazard, water logging, solute transport	Yes	Yes	Yes	No	No	No	No	Yes	Yes	[114]
MIKE-SHE	Systeme Hydrologique Europeen (French acronym for “European Hydrologic System”)	Hillslope/ Catchment	Event	High	Erosion and sediment yield	Yes	Yes	Yes	Yes	Yes	Yes	No	Yes	No	[66,115]
WESP	Watershed Erosion Prediction Project	Small Catchment	Event	Medium	Erosion, sediment yield, runoff	Yes	Yes	Yes	Yes	Yes	Yes	No	Yes	No	[116]
SEM	Soil Erosion and Sediment Transport Model	Catchment	Event	High	Erosion, sediment yield	Yes	Yes	Yes	Yes	Yes	Yes	No	No	No	[117]
SHESED	SHE- SEDimentation	Hillslope/ Catchment	Event	High	Erosion, sediment yield, runoff	Yes	Yes	Yes	No	No	No	No	Yes	No	[118]
ARMSED	Army Multiple Watershed Storm Water and Sediment Runoff	Small Catchment	Event	High	Erosion, sediment yield, runoff	Yes	Yes	Yes	No	No	No	No	Yes	No	[119]
RUNOFF	No acronym	Small Catchment	Event	Low	Erosion, sediment yield, runoff, peak rate	Yes	Yes	Yes	No	No	No	No	Yes	No	[120]
KINEROS	Kinematic Runoff and Erosion Model	Hillslope/ Small Catchment	Event	High	Erosion, sediment yield, runoff, peak rate	Yes	Yes	Yes	No	No	No	No	No	No	[121]
WEPP	Watershed Erosion Prediction Project	Hillslope/ Catchment	Daily	High	Erosion, sediment yield, runoff	Yes	Yes	Yes	Yes	Yes	Yes	No	Yes	No	[122]
PERFECT	Productivity, Erosion and Runoff, Functions to Evaluate Conservation Techniques	Field	Daily	High	Erosion, runoff, crop yield	No	No	No	No	No	No	No	Yes	Yes	[123]
OPUS	No acronym	Field/ Small Catchment	Daily	High	Erosion, suspended sediments, runoff, nutrient	Yes	Yes	Yes	No	No	No	No	Yes	Yes	[124]
GLEAMS	Groundwater Loading Effects of Agricultural Management Systems	Field	Daily	High	Erosion, sediment yield	Yes	Yes	Yes	No	No	No	No	Yes	Yes	[125]
PEPP	Process-oriented Erosion Prediction Program	Hillslope/ small catchment	Event	High	Erosion, deposition and sediment and phosphorous transport	Yes	Yes	Yes	Yes	Yes	Yes	No	Yes	Yes	[126]
CSEP	Climatic Index for Soil Erosion Potential	Regional/ Hillslope	Monthly/ Event	High	Sediment yield, runoff	Yes	Yes	Yes	Yes	Yes	Yes	Yes	Yes	No	[127]
EROSION-3D	EROSION-3D	Field/ small catchment	Event	High	Sediments dynamics	Yes	Yes	Yes	Yes	Yes	Yes	Yes	No	Yes	[128]
HEM	Hillslope Erosion Model	Hillslope	Event	High	Erosion, sediment yield, sediment concentration, runoff	Yes	Yes	Yes	Yes	Yes	Yes	No	Yes	Yes	[102]
LISEM	Limburg Soil Erosion Model	Small catchment	Event	High	Sediment yield, runoff	Yes	No	No	Yes	Yes	Yes	No	Yes	Yes	[129]
SHETRAN	European Distributed Basin Flow and Transport Modelling System	Plot/ catchment	Event	High	Sediment yield, erosion/ deposition, pollutants transport	Yes	Yes	Yes	Yes	Yes	Yes	No	Yes	Yes	[130]
GUEST	Griffiths University Erosion System Template	Plot	Steady State	High	Suspended sediment, runoff	Yes	Yes	Yes	No	No	No	No	Yes	No	[131]
SIMWE	SIMulation of Water Erosion	Catchment	Event	High	Erosion, sediment transport and deposition, gully formation	Yes	Yes	Yes	No	No	No	Yes	Yes	No	[132]
EUROSEM	European Soil Erosion Model	Catchment	Event	High	Erosion, sediment yield, runoff	Yes	Yes	Yes	No	No	No	No	No	No	[133]
EUROWISE	EUROpe WIthin Storm Erosion	Catchment	Event	Medium	Erosion, sediment yield, runoff, gully formation	Yes	No	No	Yes	Yes	Yes	Yes	Yes	No	[134]
MIKE-11	Mike (named partially after the author Michael, Mike Abbott)	Catchment	Daily	High	Sediment yield, runoff	No	No	No	Yes	Yes	Yes	No	Yes	Yes	[135]
EGEM	Ephemeral Gully Erosion Model	Field	Event	Medium	Gully formation	No	No	No	No	No	No	Yes	No	No	[136]
SEMMED	Soil Erosion Model for Mediterranean Areas	Regional	Annual	Medium	Erosion, deposition and sediment transport	Yes	Yes	Yes	No	No	No	Yes	Yes	No	[137]
CASC2D-SED	CASCade 2-Dimentional SEDimentation	Catchment	Event	High	Sediment yield, erosion/ deposition	Yes	Yes	Yes	Yes	Yes	Yes	No	Yes	No	[138]
WATEM	Water and Tillage Erosion Model	Field	Abstract	Low	Erosion, deposition and sediment transport	Yes	Yes	Yes	Yes	Yes	Yes	No	No	No	[139]
PESERA	Pan-European Soil Erosion Risk Assessment model	Regional	Annual	Medium	Erosion, runoff	Yes	Yes	Yes	No	No	No	No	Yes	No	[140]
CHILD	Channel-Hillslope Integrated Landscape Development	Hillslope/ catchment	Event	High	Erosion, sediment yield, runoff, gully formation	No	No	No	Yes	Yes	Yes	Yes	Yes	No	[141]
MWISED	Modelling Within-Storm Sediment Dynamics	Field	Event	High	Erosion, sediment yield, gully formation	No	Yes	No	Yes	Yes	Yes	Yes	No	No	[142]
KINEROS2	Kinematic Runoff and Erosion Model-2	Catchment	Event	High	Erosion, sediment yield, runoff, peak rate	Yes	Yes	Yes	No	No	No	No	Yes	No	[143]
GSSHA	Gridded Surface/ Subsurface Hydrologic Analysis	Catchment	Event	Medium	Overland erosion, sediment transport, detachment, raindrop impact, and deposition	Yes	Yes	Yes	Yes	Yes	Yes	Yes	Yes	No	[144]
DWSM	Dynamic Watershed Simulation Model	Hillslope/ catchment	Event	High	erosion, sediment yield, surface and underground runoff, flood agrochemical transport	Yes	Yes	Yes	Yes	Yes	Yes	Yes	No	Yes	[145]
REGEM	Revised Ephemeral Gully Erosion Model	Catchment	Event	High	Erosion, sediment yield, runoff, gully formation	Yes	Yes	Yes	Yes	Yes	Yes	Yes	Yes	Yes	[146]
SWAT-WB	Soil and Water Assessment Tool-Water Balance	Regional	Daily	Medium	Erosion, sediment yield, runoff, peak rate, nutrient	Yes	Yes	Yes	Yes	Yes	Yes	No	Yes	Yes	[147]

Note: Gen. * = Generation; Trans. * = Transportation; Dep. * = Deposition.

**Table 4:** Hybrid soil erosion models.

Model	Name	Spatial Scale	Temporal Scale	Data Demand	Output	Overland Sediment			In-Stream Sediment			Gully Erosion	Rainfall-Runoff	Sediment-Associated Chemicals	Source
						Gen. *	Trans. *	Dep. *	Gen. *	Trans. *	Dep. *				
MMMF	Modified Morgan, Morgan and Finney	Hillslope	Annual	High	Erosion, sediment yield and distribution, runoff	Yes	Yes	Yes	No	No	No	Yes	Yes	No	[148]
THORNES	Thornes model	Hillslope/Catchment	Event	Medium	Erosion, runoff	Yes	Yes	Yes	No	No	No	No	Yes	No	[149]
AQUALM	Networked Storm Water Quality Model	Small Catchments	Daily	Medium	Suspended sediments, runoff and pollutant transport	No	No	No	Yes	Yes	No	No	Yes	Yes	[150]
USPED	Unit Stream Power-based Erosion Deposition	Hillslope	Annual/Event	Medium	Erosion	Yes	Yes	Yes	No	No	No	No	No	No	[20]
IHACRES-WQ	Identification of unit Hydrographs and Component flows from Rainfall, Evaporation and Streamflow-Water Quality	Catchment	Daily	Low	Erosion, sediment yield, runoff, nutrients	no	no	no	Yes	Yes	Yes	Yes	Yes	Yes	[24,151,152]
SEDNET	Sediment river network model	Regional/Catchment	Annual	High	Sediment load and distribution, suspended sediments, runoff, nutrients	Yes	Yes	Yes	Yes	Yes	Yes	Yes	Yes	No	[153]
SPL	Stream Power Law Model	Catchment	Annual	Medium	Riverine erosion	Yes	No	No	No	No	No	No	No	No	[154]
SEAGIS	Erosion Assessment Tool of MIKE BASIN & MILW	Catchment	Annual	High	Erosion, sediment yield	Yes	Yes	Yes	Yes	Yes	Yes	No	Yes	No	[155]
AGWA	Automated Geospatial Watershed Assessment	Catchment	Daily/Monthly	High	Erosion, sediment yield, runoff, peak rate, nutrients	Yes	No	No	Yes	No	No	No	Yes	Yes	[19]

Note: Gen. * = Generation; Trans. * = Transportation; Dep. * = Deposition.
